# May Antitransglutaminase Levels Predict Severity of Duodenal Lesions in Adults with Celiac Disease?

**DOI:** 10.3390/medicina57111212

**Published:** 2021-11-05

**Authors:** Giuseppe Losurdo, Milena Di Leo, Edoardo Santamato, Antonio Giangaspero, Maria Rendina, Carmelo Luigiano, Enzo Ierardi, Alfredo Di Leo

**Affiliations:** 1 Section of Gastroenterology, Department of Emergency and Organ Transplantation, University “Aldo Moro” of Bari, 70124 Bari, Italy; e.santamato@studenti.uniba.it (E.S.); antoniogiangaspero@libero.it (A.G.); mariarendina@virgilio.it (M.R.); ierardi.enzo@gmail.com (E.I.); alfredo.dileo@uniba.it (A.D.L.); 2 Ph.D. Course in Organs and Tissues Transplantation and Cellular Therapies, Department of Emergency and Organ Transplantation, University “Aldo Moro” of Bari, 70124 Bari, Italy; 3 Unit of Digestive Endoscopy, San Paolo Hospital, 20142 Milano, Italy; dileomilena1984@gmail.com (M.D.L.); carmeluigiano@libero.it (C.L.)

**Keywords:** celiac disease, serology, villous atrophy, serology, anti-transglutaminase, adults

## Abstract

*Background and Objective*: Pediatric guidelines on celiac disease (CD) state that children with anti-transglutaminase antibodies (TGAs) >×10 upper limit of normal (ULN) may avoid endoscopy and biopsy. We aimed to evaluate whether these criteria may be suitable for villous atrophy diagnosis in CD adults. *Materials and Methods*: We retrospectively enrolled patients with CD aged >18 years. TGAs were expressed as xULN. Duodenal lesions were classified as atrophic or non-atrophic according to Marsh-Oberhuber. Fisher’s exact and *t*-test were used for variables comparison. Receiver operating characteristics (ROC) curve analysis was performed with estimation of area under the curve (AUC), sensitivity, specificity, and positive and negative predictive value (PPV/NPV). *Results:* One hundred and twenty-one patients were recruited. Sixty patients (49.6%) had TGA >×10 ULN, and 93 (76.8%) had villous atrophy. The cut-off of >×10 ULN had sensitivity = 53.7%, specificity = 64.3%, PPV = 83.3%, and NPV = 29.5% to predict atrophy. Therefore, considering pediatric criteria, in 50 (41.3%) patients, biopsy could have been avoided. Patient subgroup with atrophy had higher TGA levels despite being not significant (37.2 ± 15.3 vs. 8.0 ± 1.3 ULN, *p* = 0.06). In adults, a slightly better diagnostic performance was obtained using a cut-off of TGA >×6.2 ULN (sensitivity = 57.1%, specificity = 65.6%, and AUC = 0.62). *Conclusions:* Despite our confirmation that villous atrophy is linked to high TGA levels, CD and atrophy diagnosis based only on serology is not reliable in adults.

## 1. Introduction

Celiac disease (CD) is an autoimmune disorder characterized by small intestinal enteropathy caused by gluten ingestion in genetically predisposed individuals. Gluten is able to elicit an autoimmune response that leads to lymphocyte infiltration in duodenal mucosa, thus causing inflammatory damage and villous atrophy in the long run [1]. Symptoms are often vague and not specific. The diagnosis is achieved by the detection of specific autoantibodies (serology) and/or villous atrophy in endoscopic duodenal biopsy samples. In pediatric population, European Society of Pediatric Gastroenterology, Hepatology, and Nutrition (EPSGHAN) guidelines (2012, revised on 2020) approved current criteria for the diagnosis: biopsy could be avoided when anti-transglutaminase antibodies (TGAs) value exceeded the cut-off over ×10 upper limit of normal (ULN) and anti-endomysium antibodies (EMA) were positive, independently from value [2].

Conversely, the diagnosis of CD in adult patients requires the simultaneous assessment of serology and duodenal biopsies taken during upper gastrointestinal endoscopy, although in Finland, national guidelines state that diagnosis can be made without biopsy in adults when TGAs are over ×10 ULN, and anti-endomysium is positive [3]. However, at least the 10% of specimens may not have an acceptable quality due to insufficient size or lack of orientation, and sometimes, endoscopy needs to be repeated [4]. Moreover, endoscopy is an invasive procedure with risk of complication, it is expensive, and sedation is often required.

The promising data found in pediatric literature [2] have, therefore, pushed investigations in order to ascertain whether a pure serologic approach may be used in adults with suspicion of CD.

Several studies supported the “no-biopsy strategy” in adult population [5,6] since they found that IgA TGA ×10 ULN could be used as diagnostic cut-off also in adult population, with sensitivity and specificity close to 100% [7,8]. Other reports stated that cut-off values of antibody TGA levels [9,10,11,12,13] ranging from ×7 ULN to ×16 ULN reflected villous atrophy.

Based on the lack of clear evidence, the aim of the present study was to provide further data about the possibility that positive serology (anti-transglutaminase level ≥10× ULN and anti-endomysium positivity) could be adequate for the diagnosis of villous atrophy in CD adult population.

## 2. Materials and Methods

### 2.1. Patients

We carried out a retrospective cohort study, including data collected in a prospectively maintained database of Gastroenterology Unit of Policlinico (Bari, Italy), from 1999 to 2020. The study was reviewed and approved by the Ethics Committee Board of University Hospital Policlinico of Bari (protocol No. 11907/11/02/2021).

All enrolled patients fulfilled the following criteria: (i) adult age (>17 years old), (ii) serology test for CD available in records, and (iii) upper gastrointestinal endoscopy with duodenal biopsy sampling.

Exclusion criteria were: (i) serology performed more than 12 weeks from endoscopy, (ii) previous diagnosis of CD, (iii) previous gluten-free diet (GFD) in the month before serology and/or endoscopy, and (iv) selective IgA deficiency or other immunoglobulin deficiency.

### 2.2. Serology and Other Laboratory Investigations

Patients were tested for IgA anti-transglutaminase (TGA), anti-endomysium (EMA), and anti-deamidated gliadin peptides (DGP). In particular, TGA levels were expressed as IU/mL and as upper limit of normal (ULN), i.e., as a ratio with the threshold for positivity indicated by the laboratory.

The following variables were collected: hemoglobin, albumin, vitamin B12, iron, transferrin, vitamin D, and vitamin B12, if available.

If needed, HLA testing was performed, and the result was collected.

In all patients, clinical variables were also collected as age, symptoms, and familial history for CD.

### 2.3. Endoscopic Procedures

During upper gastrointestinal endoscopy, at least four duodenal biopsies were performed, including 1–2 biopsies from the bulb.

The biopsy was performed one bite per pass of the forceps [14], and the specimen was oriented and stored in 10% neutral buffered formalin. Histological diagnosis was reported according to Marsh classification [15].

### 2.4. Outcomes

The primary outcome was to evaluate sensibility, specificity, positive predictive value (PPV), and negative predictive value (NPV) of TGA titer of ≥10× ULN plus EMA positivity to diagnose villous atrophy in CD patients. The secondary outcomes of the study were the identification of an optimal TGA titer cut-off for villous atrophy diagnosis in CD adult population and the estimation of the number of cases in which such no-biopsy approach could avoid endoscopy in adults.

### 2.5. Statistical Analysis

Continuous data were reported as either mean and standard deviation (SD). They were compared using Student’s *t*-test. Categorical data were described as percentage and compared using chi-square test. The diagnostic performance of TGA titer ≥10× ULN was determined by calculating the sensitivity, specificity, and positive and negative predictive values considering level 3 according to Marsh classification as gold standard for villous atrophy diagnosis in CD comparing Marsh classification level <2.

Receiver operating characteristic (ROC) curves were drawn, and area under the curve (AUC) was calculated to determine the optimal cut-off value of TGA titer to increase accuracy of TGA titer at predicting villous atrophy diagnosis in CD.

A sample size of 121 patients was estimated based on the following assumptions:

(a) Previously reported sensitivity and specificity of TGA titer ≥10× ULN were 54% and 90%, respectively [16],
(b) 1% estimated prevalence of CD, 90% precision, and 90% confidence interval, and
(c) A *p*-value < 0.05 was considered statistically significant. Statistical analysis was performed using SPSS 23 for Windows.

## 3. Results

### 3.1. Patients Characteristics

**Figure 1:**
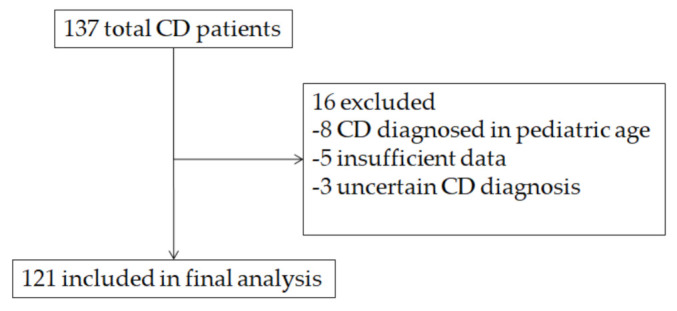
Flowchart of patients selection.

One hundred and thirty-eight patients records were screened in our database. However, only 121 met inclusion and exclusion criteria since in 8 of them, CD diagnosis was achieved in pediatric age; in three, the diagnosis was not certain; and data for statistical analysis were lacking in five patients (Figure 1).

Twenty-five (20.7%) were male, while 96 were female. Their mean age was 34.7 ± 12.3 years (range 19–73), while the mean age at CD diagnosis was 34.7 ± 12.3 (range 18–72). The most common symptoms at diagnosis were weight loss (27.3%), abdominal pain (49.4%), diarrhea (33.1%), and anemia (44.6%). All patients were DQ2/8 carrier, and nine of them in a homozygosis state. Forty patients (33.1%) showed association with at least one autoimmune disease, the most common ones being autoimmune thyroiditis (25 patients, 20.7%) and dermatitis herpetiformis (14 subjects, 11.6%). No patient had IgA deficiency or other immunoglobulin deficit. Further details are reported in Table 1.

### 3.2. TGA Levels in Villous Atrophy Patients

Villous atrophy was detected in 93 (76.8%) subjects at duodenal biopsy samples. The remaining 28 patients had a Marsh 1 or 2 stage. When comparing patients with versus without villous atrophy (Table 2), no difference regarding demographic features, clinical symptoms, or iron and other vitamin status emerged. However, TGA levels were slightly higher in patients with villous atrophy, despite this comparison being close to significance (378.5 ± 1480.7 vs. 100.9 ± 95 IU/mL, *p* = 0.08). Similarly, this trend was retained even when TGA were normalized for upper limit range (37.2 ± 148.1 vs. 8.0 ± 7.1, *p* = 0.06). Positivity rate of EMA and DGP did not differ between the two groups. However, all patients with TGA >×10 ULN were EMA positive.

### 3.3. Cutoffs for Serological Diagnosis

**Figure 2:**
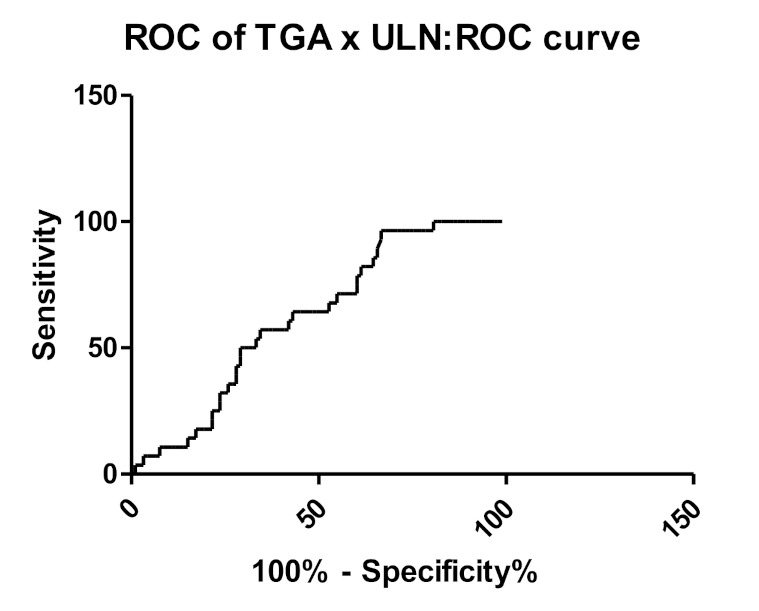
Receiver operating characteristic (ROC) curve referring to TGA normalized per ULN.

Considering the ESPGHAN criteria for a no-biopsy diagnosis, the ×10 ULN cutoff had a sensitivity of 53.7%, specificity of 64.3%, PPV of 83.3%, and NPV of 29.5%. This cut off could avoid biopsy sampling in 50 (41.3%) patients. Instead, the same cut off had a sensitivity of 51.7%, specificity of 100%, PPV of 100%, and NPV of 91.8% for diagnosis of Marsh ≥2. ROC curve (Reported in Figure 2) found that TGA ×6.2 ULN had the best diagnostic performance for villous atrophy, with a sensitivity of 57.14%, a specificity of 65.59%, a PPV of 82.4%, a NPV of 31.9%, and an AUC of 0.62 ± 0.05. Such cutoff could allow to avoid biopsy in 61 (50.4%) subjects. The results of ROC curve analysis are summarized in Table 3.

## 4. Discussion

Even though ESPGHAN guidelines allow to avoid duodenal biopsy sampling in children with TGA > ×10 ULN, EMA positivity, and clinical suspicion for CD diagnosis [2], adults guidelines still recommend an integration of serologic results with histological picture [17,18]. Nevertheless, several attempts to investigate whether the ESPGHAN approach could be suitable for adults have been performed.

Some studies have confirmed the optimal effectiveness of ×10 ULN threshold in adult population. Hill [19] found that such value had a 100% positive predictive value since all patients with TGA above such range had typical histological lesions. However, in this study, only the 58% of 112 CD subjects had TGA > ×10 ULN, thus limiting the wide application of this threshold. In the recent study by Penny et al. [16], while specificity and positive predictive value were high (90% and 98.7%, respectively), sensitivity and negative predictive values were disappointing (54% and 12.5%, respectively). The high specificity could be a hint that villous atrophy is unlikely when TGAs are low. Meanwhile, the low sensitivity may suggest that high TGA is not adequate to achieve diagnosis.

On the other hand, other studies have been focused on discovering cut-off values less than ×10 ULN with a sufficiently high specificity and/or PPV for an exclusive serological diagnosis. Among these, Tortora [20] conducted a prospective observational study to determine which was the best TGA cut-off to predict a Marsh 2 (minimum level required to diagnose celiac disease) and a Marsh 3 (corresponding to villous atrophy). The best threshold predicting Marsh 2 was ×6.4 ULN (with a PPV and a specificity of 100% and a sensitivity of 70%), while ×8.9 ULN showed sensitivity of 69% and specificity of 100% for Marsh 3. In another Italian multicenter retrospective analysis, similar results were found even for a TGA value > ×7 ULN [21]. Finally, Di Tola et al. calculated that a TGA value of ×3.6 ULN would provide sensitivity of about 75% and a PPV close to 100% [22].

Our study has not confirmed that patients with advanced mucosal lesions have high TGA titer, as already described in the literature [23]; however, we found a non-statistically significant trend of higher TGA title in patients with advanced mucosal lesions. Additionally, we found that the ×10 cut-off had a PPV of 83.3%, specificity of 64.3%, and sensitivity of 53.7%. These values are clearly lower than those from similar experiences even if they confirm that specificity of serology is better than sensitivity. On the other hand, the same cut-off was more efficient to diagnose a Marsh of 2 or higher, with PPV of 100%, which is comparable to other evidence [8,9].

Furthermore, we investigated whether other cut-off values could work with better sensitivity and specificity and found that the cut-off of ×6.2 ULN had a sensitivity of 57% and a specificity of 65.59% (both higher than ×10 ULN); the AUC of the ROC curve was equal to 0.62, which is still a suboptimal value.

The present work could have some pros that should be acknowledged. The sample size (121 patients) is adequate and representative of multiple aspects of sex, age, comorbidities, and clinical symptoms. Our cohort indeed confirmed a high variability of extraintestinal manifestations, as already described in literature [24]. Furthermore, it is a real-life study that involves patients followed up at the celiac outpatient clinic of our Centre in Southern Italy. We collected data of a large pool of patients from all over our region (Puglia), enriched by more of 20 different variables. Since the study was retrospective, data deriving from laboratory and endoscopic examinations performed at different centers were analyzed: this implies the use of different reference values or different detection methods that necessarily may create heterogeneity for statistical analysis, and this could be a major concern. To overcome this possible limit, we considered the normalized variables with respect to the ULN for TGA. Nevertheless, the histological interpretation of the sample could not be uniform, as it was conducted by different pathologists. Additionally, it is known that guidelines recommend performing at least four biopsy samples in the duodenum and two in the bulb [25]; this suggestion may not have been always followed since it is time consuming and hard to be implemented by endoscopists.

Diagnostic performance in our study was markedly lower than in other ones [5], and this could be explained by the different population cohort and controls. Indeed, we enrolled only CD patients, while in other studies, patients with low probability of CD were recruited. It is obvious that, when considering patients with heartburn or dyspepsia as controls, the probability to have both atrophy and high TGA is low, and this causes an increase in sensitivity. Indeed, it has been shown that, when the pre-test probability of CD diagnosis is <10%, exclusive serological diagnosis has a low post-test probability, lower than 90% [26]. Therefore, the present study advises that the serological approach should be applied only to subjects with high suspicion of CD, as we did, and this invariably leads to lower test effectiveness.

Overall, these data suggest that the threshold for TGA in adults could be lower than in children, but the diagnostic performance is not optimal to warrant a no-biopsy approach in adult. It works more properly for Marsh 2 detection, but it is still debated whether Marsh 2 could be considered enough to confirm CD. Furthermore, it should be noted that, in adult patients, the possibility of refractory CD, neoplastic complication of CD, is more common than in children, and the no-biopsy approach could miss such conditions [27,28]. This strategy became necessary for children to avoid a troublesome endoscopic procedure, but adults are more compliant when undergoing upper endoscopy.

## 5. Conclusions

In conclusion, although some results are encouraging, the literature referring to this topic is still small. The present study confirms that the execution of duodenal biopsy samples and subsequent histological examination are still necessary to diagnose celiac disease and that serology should be integrated with histology in the decision-making process. However, further forthcoming studies should be promoted to provide potential innovative information in this complex setting.

## Figures and Tables

**Table 1:** Main features regarding enrolled population, including demographic data, clinical symptoms, serologic profile, and laboratory investigations.

Variable.	*n* (%) or Mean ± SD
Male sex	25 (20.7%)
Age (years)	38.6 ± 12.3 (range 20–73)
Age at diagnosis (years)	34.7 ± 12.3 (range 18–72)
First degree familiarity for CD	26 (21.5%)
Diarrhea	40 (33.1%)
Weight loss	33 (27.3%)
Abdominal pain	60 (49.6%)
Dyspepsia	57 (47.1%)
Iron deficiency anemia	54 (44.6%)
Autoimmune diseases	40 (33.1%)
Dermatitis herpetiformis	14 (11.6%)
DQ2/DQ8 homozygosis	9 (7.4%)
Villous atrophy	93 (76.9%)
TGA (IU/mL, n.v. 1–10)	314.3 ± 1302.6 (range 1.8–13,500)
TGA normalized ×ULN	30.5 ± 130.3 (range 0.11–1350)
EMA positivity	100 (82.6%)
DGP positivity	50 (41.3%)
Hemoglobin (g/dL, n.v. 12–15)	12.9 ± 1.8 (range 7.1–16.9)
Blood iron (mcg/dL, n.v. 50–170)	69.9 ± 42.9 (range 6.9–176)
Ferritin (mcg/dL, n.v. 8–252)	37.4 ± 54.9 (range 1–297)
Transferrin (mg/dL, n.v. 200–360)	271.1 ± 72.2 (range 190–465)
Albumin (g/dL, n.v. 3.4–5)	4.1 ± 0.4 (range 2.93–4.83)
Vitamin D (ng/mL, n.v. > 20)	22.8 ± 9.7 (range 4.2–55.7)
Vitamin B12(ng/mL, n.v. 150–650)	389.5 ± 190.1 (range 44–859)
Folate (ng/mL, n.v. 3.9–26.8)	4.7 ± 3.7 (range 1–20)

CD, celiac disease; DGP, anti-deamidated gliadin peptides antibody; EMA, anti-endomysium antibodies; n.v., normal value; SD, standard deviation; TGA, anti-transglutaminase antibodies; ULN, upper limit of normal.

**Table 2:** Comparison of main characteristics of CD patients with and without villous atrophy.

Variable	Atrophy (*n* = 93)	No Atrophy (*n* = 28)	*p*
Male sex	20 (21.5%)	5 (17.8%)	0.79
Age (years)	38.7 ± 12.6	38.1 ± 11.4	0.79
Age at diagnosis (years)	35.0 ± 12.7	33.8 ± 11.1	0.63
First degree familiarity for CD	20 (21.5%)	6 (21.4%)	1.00
Diarrhea	29 (31.2%)	11 (39.3%)	0.45
Weight loss	21 (22.6%)	12 (42.9%)	0.06
Abdominal pain	43 (46.2%)	17 (60.7%)	0.22
Dyspepsia	44 (47.3%)	13 (46.4%)	0.66
Iron deficiency anemia	42 (45.1%)	12 (42.9%)	0.93
Autoimmune diseases	32 (34.4%)	8 (28.6%)	0.65
Dermatitis herpetiformis	10 (10.8%)	4 (14.3%)	0.74
DQ2/DQ8 homozygosis	6 (6.5%)	3 (10.7%)	0.43
TGA (IU/mL)	378.5 ± 1480.7	100.9 ± 95.0	0.08
TGA normalized ×ULN	37.2 ± 148.1	8.0 ± 7.1	0.06
EMA positivity	75 (80.6%)	25 (89.3%)	0.40
DGP positivity	37 (39.8%)	13 (46.4%)	0.67
Hemoglobin (g/dL)	12.9 ± 1.7	12.7 ± 2.1	0.68
Blood iron (mcg/dL)	72.1 ± 44.9	62.4 ± 35.4	0.31
Ferritin (mcg/dL)	36.5 ± 55.8	40.5 ± 52.6	0.8
Transferrin (mg/dL)	282.3 ± 76.2	226.3 ± 28.0	0.07
Albumin (g/dL)	4.0 ± 0.4	4.3 ± 0.3	0.06
Vitamin D (ng/mL)	23.7 ± 10.1	18.8 ± 6.9	0.08
Vitamin B12 (ng/mL)	398.6 ± 188.0	358.9 ± 201.0	0.52
Folate (ng/mL)	5.0 ± 3.9	3.8 ± 3.1	0.24

CD, celiac disease; DGP, anti-deamidated gliadin peptides antibody; EMA, anti-endomysium antibodies; SD, standard deviation; TGA, anti-transglutaminase antibodies; ULN, upper limit of normal.

**Table 3:** Sensitivity, specificity, and positive and negative predictive value, area under the curve (AUC) of TGA normalized per ULN.

	TGA × ULN
Cut off	6.2
Sensitivity	57.14%
Specificity	65.59%
Positive predictive value	82.4%
Negative predictive value	31.9%
AUC	0.62
